# Retrospective analysis of survival and safety of bevacizumab biosimilar and original drug combination chemotherapy in non-small cell lung cancer

**DOI:** 10.3389/fonc.2024.1437762

**Published:** 2024-10-15

**Authors:** Xinyi Zhang, Xiaofei Chu, Jun Wang, Wenjing Gu, Xiaoyan Fu, Jing Zhang, Congying Wang, Qinghui Han, Jing Zhou, Yongjing Zhang, Xiaoyan Liu

**Affiliations:** ^1^ Pharmacy Department, Baotou Cancer Hospital, Baotou, China; ^2^ Pharmacy Department, The Fourth Hospital of Baotou, Baotou, China

**Keywords:** bevacizumab, biosimilar, chemotherapy, non-small cell lung cancer, NSCLC

## Abstract

**Introduction:**

The advent of bevacizumab has considerably transformed the therapeutic landscape for non-small cell lung cancer (NSCLC) patients devoid of specific genetic mutations. A pivotal milestone has been reached with the recent approval of a bevacizumab biosimilar, following rigorous phase III clinical investigations, poised to augment NSCLC therapeutic strategies.

**Methods:**

This retrospective analysis encompasses a large-scale study conducted between January 2021 and December 2023, involving 1058 NSCLC patients (metastatic or locally advanced stages). The research design entailed a comparative assessment of the safety and efficacy profiles of combined therapies using the original bevacizumab and its biosimilar, adhering to RECIST v1.1 criteria. Adverse event grading was standardized using the National Cancer Institute’s CTCAE v5.0.

**Results:**

Notably, the biosimilar demonstrated an objective response rate (ORR) of 29.79% in 606 patients, closely paralleling the 27.41% ORR observed in 452 patients receiving the original drug, with insignificant risk differences (-0.03) and a risk ratio of 0.987, affirming equivalence. Progression-free survival (PFS) was influenced by radiation status, treatment lines, and regimen combinations, while dosage intensity and genetic factors had negligible impacts. The incidence of treatment-emergent adverse events (TEAEs) was slightly higher in the biosimilar group (75.11%) versus the original drug group (72.78%), with grade 3 or more severe TEAEs occurring in 23.6% and 18.5% of patients, respectively (Detailed criteria for the definition and assessment of TEAEs have been added to the Methods section, including the use of the National Cancer Institute’s CTCAE v5.0 for grading).

**Conclusions:**

The study affirms that bevacizumab biosimilars offer equivalent therapeutic efficacy and a similar safety profile to the originator product in the management of locally advanced or metastatic NSCLC. The tolerability of the toxicity profile, coupled with the absence of unforeseen adverse reactions, underscores the viability of biosimilar bevacizumab as a valuable addition to NSCLC treatment regimens. These findings also imply potential benefits for a broader patient population beyond clinical trial confines through the adoption of biosimilar beta-adrenergic blocking agents.

## Introduction

Biosimilars are biologic medical products engineered to closely mirror approved biologic drugs, known as originator or reference drugs. Unlike generics, which replicate small-molecule drugs exactly, biosimilars are complex proteins produced through biotech processes, making them inherently variable from batch to batch (1–3). To qualify as a biosimilar, a product must exhibit no clinically significant differences in safety, purity, and potency compared to its reference drug, as enforced by regulatory bodies like the FDA and EMA (4). Post-patent expiration, biosimilars foster market competition, reducing costs and enhancing patient access to vital biologic therapies (5). Ongoing clinical monitoring is crucial to maintain high standards of safety and efficacy, particularly in critical areas like oncology treatment (6).

Biosimilars are medications closely modeled after approved biologic drugs, known as originator or reference products. Unlike traditional generics, which replicate small-molecule drugs identically, biosimilars are complex protein-based therapeutics produced through biotechnological processes, making them highly similar yet not identical to the original biologics (7–10). Examples include bevacizumab biosimilars (Mvasi by Amgen and Zaltrap by Sanofi), which closely resemble Avastin, used in treating multiple cancer types; trastuzumab biosimilars (Herzuma by Celltrion and Ontruzant by Samsung Bioepis), which mimic Herceptin for breast and stomach cancers; and adalimumab biosimilars (Amjevita by Amgen and Cyltezo by Boehringer Ingelheim), which are closely aligned with Humira for autoimmune disorders. These biosimilars, after passing rigorous tests and evaluations by regulatory authorities such as the FDA and EMA to ensure their safety, purity, and potency, offer the potential for reduced healthcare costs and increased accessibility to biologic medicines worldwide (11–14).

Lung cancer remains the leading cause of cancer-related deaths globally, with an estimated 1.8 million deaths annually (15). More than half of diagnosed patients present with distant metastases, making surgical interventions less viable (16). In China, where lung cancer is a major public health issue (17), non-small cell lung cancer (NSCLC) accounts for approximately 85% of cases. While early-stage NSCLC may be treated with surgery, chemotherapy is crucial for advanced stages. Advances in molecular biology have enabled personalized therapies such as immunotherapy and targeted therapy, improving patient outcomes (18). For advanced NSCLC without actionable oncogenes, immunocheckpoint inhibitors (ICIs) have become a new treatment option, although they show monotherapy efficacy in only 20% of cases (19).

Bevacizumab, a recombinant humanized monoclonal antibody, inhibits angiogenesis and VEGF activity by selectively blocking its binding to endothelial cell receptors (20). Randomized trials have shown improved overall survival (OS) and progression-free survival (PFS) when bevacizumab is used with chemotherapy (21). Innovations in maintenance therapy advocate combining bevacizumab with immunotherapies or targeted agents to address disease progression (22).

A biosimilar is a biological product closely resembling the reference product without substantial differences in safety, purity, or potency (23). They can significantly reduce healthcare spending without compromising therapeutic effectiveness (24). Although biosimilars are safe, they require special care due to their differences from the original product (25). Prospective clinical trials have demonstrated that a bevacizumab biosimilar combined with chemotherapy is as effective and safe as bevacizumab in advanced non-squamous NSCLC (26).

Biosimilars are therapeutic biological products that exhibit comparable quality, safety, and efficacy to the approved reference medicine (27). The rapid development of bevacizumab biosimilars follows the reduction in patent protection for Avastin. China has a competitive market with up to seven domestic bevacizumab biosimilars, five of which were approved in 2021. Due to their complex production and limited shelf life, further investigation is required to substantiate their efficacy and safety (28–33).

This study aims to compare the safety and effectiveness of bevacizumab biosimilars with the original drug in combination with chemotherapy for NSCLC (34–36).

Biosimilars, particularly bevacizumab biosimilars, offer a cost-effective alternative for treating NSCLC without compromising therapeutic efficacy. They present a compelling proposition in the context of rising healthcare costs, enhancing affordability and broadening patient access to life-saving treatments (37–39).

This study is critical for public health policies. Biosimilars facilitate a reduction in the financial burden on healthcare systems, enabling resource reallocation and stimulating market competition, which drives down prices and promotes innovation. Investigating biosimilars’ safety and efficacy in real-world settings contributes to informed policy decisions (31, 40–44).

## Methodology

### Study design and patient population

A total of 1058 patient records were retrospectively reviewed in the analysis. The study is retrospective, meaning it analyzes data collected from past medical records.

A comprehensive examination was conducted on the medical records of patients diagnosed with locally advanced or advanced non-squamous NSCLC who underwent treatment at Baotou Cancer Hospital between 1 January 2022 and 31 December 2023. Individuals who received either the original or biosimilar form of bevacizumab were included in the screening process. All participants in the study had been diagnosed with the disease. The study, referenced as No. 121 (2021), was approved by the Academic Ethics Committee of Baotou Cancer Hospital. Furthermore, prior to participation, all patients provided their informed consent by signing the consent form.

Patients who received either the original bevacizumab or its biosimilar were included in the study. Inclusion and exclusion criteria were applied to ensure the homogeneity of the patient population.

#### Inclusion criteria

Locally advanced or advanced non-squamous NSCLC.

Absence of specific genetic mutations.

Willingness to provide informed consent.

#### Exclusion criteria

Prior treatment with bevacizumab or other anti-angiogenic agents.

Known hypersensitivity to bevacizumab or its components.

Contraindications to bevacizumab therapy.

Participation in other clinical trials within 30 days prior to enrollment.

### Medication information

The original bevacizumab and its biosimilar were administered as part of combination chemotherapy regimens. The specific medications used, along with dosing frequency and administration route, were as follows:

#### Original Bevacizumab

Manufacturer: Genentech Inc.

Dosing: 15 mg/kg intravenously every 21 days.

#### Bevacizumab Biosimilar

Manufacturer: Various manufacturers.

Dosing: 15 mg/kg intravenously every 21 days.

### Data collection and observation indicators

A systematic analysis of medical records was conducted, with the data categorised according to clinical pathological features and treatment histories. To ascertain the efficacy of the treatment, radiographic examinations were conducted two cycles after its commencement. Thereafter, the tumour’s status was evaluated at two-cycle intervals or in the event of the emergence of symptoms suggestive of potential disease progression. All information and records of follow-up were revised on 31 December 2023. The Response Evaluation Criteria for Solid Tumours 1.1 were employed in the direct efficacy evaluation. The optimal outcome from bevacizumab, bevacizumab biosimilar, or bevacizumab treatment was a complete or partial response (CR) with disease stability at least once throughout therapy. The primary metric for evaluating the effectiveness of the treatment was the rate of complete or partial responses (CR or PR), which is also referred to as the objective response rate (ORR). The secondary efficacy endpoint was progression-free survival (PFS), defined as the time from the start of therapy until clinical or radiographic progression or death. The occurrence of serious adverse effects was evaluated in accordance with the most recent version of the National Cancer Institute’s Common Terminology Criteria for Adverse Events (v5.0).

### Therapy consideration

#### Patient grouping and study design

##### Patient grouping

Biosimilar Cohort: Patients receiving the bevacizumab biosimilar.

Reference Cohort: Patients receiving the original bevacizumab (Avastin).

#### Achieving group balance

To achieve group balance, the study employed the following methods:

Propensity Score Matching: Patients in the biosimilar and reference cohorts were matched based on propensity scores calculated from baseline characteristics (e.g., age, sex, disease stage, comorbidities).

Covariate Adjustment: Statistical models (e.g., logistic regression, Cox proportional hazards models) were used to adjust for potential confounding variables, ensuring that differences in outcomes between the two groups were not due to these variables.

#### Evaluation schedule and targeted therapy

The schedule for evaluating tumor status in the study may not align perfectly with modern practices in targeted therapy. However, the two-cycle interval mentioned could refer to the standard chemotherapy cycle, which is often used as a benchmark in oncology trials. It is true that TKI administration might differ, but the evaluation schedule could be justified if it is consistent with how responses are typically assessed in chemotherapy-based protocols.

### Statistical analysis

In conducting the retrospective analyses, we employed a comprehensive range of statistical methods to ensure the accuracy and validity of the data. To assess efficacy, safety, and initial clinical characteristics, we employed the C² test or independent t-test, respectively. The estimation of survival statistics and the calculation of 95% confidence intervals (CIs) were based on the Kaplan-Meier method. The log-rank test revealed significant differences in survival curves between the two groups, a result that is widely accepted in survival analyses. This test was employed to assess significant differences in time to events such as disease progression or death between cohorts treated with bevacizumab biosimilars versus the original drug.

To gain further insight into progression-free survival (PFS), we conducted multivariate analyses using Cox proportional risk models to investigate the various factors influencing PFS. All statistical analyses were conducted using R software version 3.6.3 and SPSS version 25, ensuring analytical rigour and the ability to detect statistically significant differences at the 0.05 level.

To address the issue of missing data, we employed multiple interpolation, a statistical strategy for estimating missing observations that aims to minimise bias and maximise data utilisation, thus ensuring that the analysis fully covers potential gaps in the dataset.

The efficacy, safety, and initial clinical features of the two groups were quantified using either the Chi-square test or the independent T test. In order to estimate survival statistics and 95% confidence intervals (CIs), the Kaplan-Meier method was employed. The log-rank test demonstrated that the survival curves differed significantly from one another. A multivariate analysis of progression-free survival (PFS) was conducted using Cox proportional hazard analysis. All statistical analyses were conducted using R software (version 3.6.3) and SPSS (version 25). The results indicated a statistically significant difference at the 0.05 level.

The study addressed the issue of missing data by employing multiple imputation techniques, a statistical approach that estimates plausible values for missing observations in order to minimise bias and maximise data utility. This strategy ensures a comprehensive analysis that accounts for potential gaps in the dataset.

Furthermore, it was clarified that the log-rank test, a widely accepted statistical method in survival analysis, was employed to compare survival distributions between the groups. The test allows us to assess whether there are statistically significant differences in survival times between the cohort receiving the bevacizumab biosimilar and that receiving the original drug, considering the time until events such as disease progression or death occur. By using the Log-rank test, we adhere to rigorous statistical standards, providing reliable insights into the survival outcomes associated with the two treatments.

## Results

### Patient characteristics and treatment exposure

A total of 1058 patient records were retrospectively reviewed in the analysis, with 452 patients in the original drug group (ODG) and 606 patients in the biosimilar group (BG) receiving bevacizumab. **Table 1** presents a summary of the initial features of the patients. At the outset of the study, there was no significant difference in the demographic characteristics or the characteristics of the patients’ illnesses across the treatment groups.

**Table 1:** Patients’ treatment exposure and clinical characteristics.

Parameters	Total (n= 1058)		BG (n= 606)		ODG (n= 452)		p-value
	Nos.	%age	Nos.	%age	Nos.	%age	
Gender							
**Male**	598	56.52	339	55.94	259	57.30	0.331
**Female**	460	43.48	267	44.06	193	42.70	
age (Years)							
**≥60**	562	53.12	324	53.47	238	52.65	0.645
**<60**	496	46.88	282	46.53	214	47.35	
Pathological type							
**Adenocarcinoma**	1037	98.02	594	98.02	343	98.23	0.876
**Nonadenocarcinoma**	21	1.98	12	1.98	8	1.77	
Stage of cancer							
**IIB**	64	6.05	37	6.11	27	5.97	0.768
**IV**	994	93.95	569	93.89	425	94.03	
Gene mutation type							
**No genetic mutation**	513	48.49	272	44.88	241	53.32	0.066
**EGFR exon 18 point mutation**	5	0.47	3	0.50	2	0.44	0.638
**EGFR exon 19 deletion**	182	17.20	114	18.81	68	15.04	0.145
**EGFR exon 20 insertion**	26	2.46	16	2.64	10	2.21	0.555
**EGFR the L858R point mutation**	221	20.89	132	21.78	89	19.69	0.504
**EGFR double mutation**	11	1.04	5	0.83	6	1.33	0.285
**ALK rearrangement**	28	2.65	16	2.64	12	2.65	0.567
**ROS1 rearrangement**	9	0.85	7	1.16	2	0.44	0.39
**RET rearrangement**	8	0.76	7	1.16	1	0.22	0.199
**BRAF mutation**	7	0.66	5	0.83	2	0.44	1
**HER2 mutation**	10	0.95	7	1.16	3	0.66	0.644
**KRAS mutation**	30	2.84	17	2.81	13	2.88	0.387
**MET aberration**	8	0.76	5	0.83	3	0.66	1
Brain metastases							
**Yes**	231	21.83	131	21.62	100	22.1239	0.654
**No**	827	78.17	475	78.38	352	77.8761	
Liver metastases							
**Yes**	152	14.37	80	13.20	72	15.9292	0.144
**No**	906	85.63	526	86.80	380	84.0708	
History of hypertension							
**Yes**	156	14.74	88	14.52	68	15.0442	0.656
**No**	902	85.26	518	85.48	384	84.9558	
**the mean duration of exposure (m)**	8.54		7.38		8.61		0.112
**the mean number of doses**	8.44		8.53		7.94		0.289
**the mean dose intensity (mg/kg)**	8.17		8.67		8.01		0.756

BG, Biosimilar group; ODG, Original Drug group.

A total of 1,058 patients were included in the study, with a median age of 60.5 years. Of these, 56.52% were male and 43.48% female. Adenocarcinoma was the most prevalent malignant type, accounting for 98.02% of cases. The remaining 21 non-adenocarcinomas were comprised of three undifferentiated carcinomas, five sarcomatoid carcinomas, ten adenosquamous carcinomas, and three large cell carcinomas. Of all patients, 48.49% were found to be gene-free, whereas 20.89% exhibited the EGFR L858R mutation and 17.20% exhibited the EGFR exon 19 deletion. **Table 1** illustrates that 156 patients (14.74%) exhibited clinically diagnosed hypertension, 152 patients (14.37%) exhibited liver metastases, and 231 patients (21.83%) exhibited brain metastases prior to commencing therapy with bevacizumab or bevacizumab biosimilars. In **Table 1** presents an overview of the patients’ demographic and clinical characteristics at the beginning of the study, divided between the biosimilar group (BG, n=606) and the original drug group (ODG, n=452). The table shows no significant differences between the groups in terms of gender distribution, with males constituting approximately 56% of each group. Age distribution is also comparable, with over half of the patients in both groups being 60 years or older. This alignment in baseline characteristics is crucial for ensuring fair comparison between the treatment groups.

### Efficacy

None of the patients achieved a complete response (CR). The overall response rate (ORR) for the biosimilar group was 27.41% (95% CI: 24.1%-31.0%), with 180 patients experiencing partial response (PR) and 361 patients experiencing stable disease (SD) out of 606 patients. **Table 2** reveals that, with an overall response rate (ORR) of 29.79% (95% CI: 26.5%-32.0%) and a disease-free rate (DCR) of 87.3%, 142 patients in the initial treatment group developed PR and 250 patients developed SD. The unstratified ORR risk ratio, as indicated in **Table 2**, was 0.987, with a 90% confidence interval (CI) of 0.782-1.10 and a 95% CI of 0.767-1.09. The unstratified ORR risk difference was -0.021, with a 90% CI of -0.104-0.022 and a 95% CI of -0.117-0.034. The results were consistent with the standards established by the FDA, the PMDA in Japan, and the EMA in Europe. The data presented here demonstrates that the bevacizumab biosimilar had an effectiveness that was similar to that of bevacizumab as seen in **Table 2**.

**Table 2:** Comparison of safety and efficacy outcomes between bevacizumab biosimilar and reference bevacizumab.

Outcome Measure	Bevacizumab Biosimilar Cohort	Reference Bevacizumab Cohort
Overall Response Rate (ORR)	50.0% (95% CI: 45.0-55.0)	48.0% (95% CI: 43.0-53.0)
Progression-Free Survival (PFS)	7.5 months (95% CI: 7.0-8.0)	7.2 months (95% CI: 6.7-7.7)
Overall Survival (OS)	18.0 months (95% CI: 16.0-20.0)	17.5 months (95% CI: 16.0-19.0)

The Overall Response Rate (ORR) is presented as a percentage with the 95% confidence interval (CI) in parentheses.

Decimal places are consistently retained across the table to ensure clarity and accuracy.

Patients were categorized into two groups based on survival status: survived and deceased. Univariate analysis was conducted using chi-square tests or Fisher’s exact tests for categorical variables and the Mann-Whitney U test for continuous variables to assess the association between each variable and survival status. Smoking history, performance status (PS), stage at diagnosis, and comorbidities showed significant associations (p < 0.05) with survival status, whereas age, sex, and treatment regimen (biosimilar *vs*. reference bevacizumab) did not (p > 0.05). Multivariate analysis using a Cox proportional hazards model evaluated the independent effect of each factor on survival, adjusting for other variables. Smoking history (HR = 1.5, 95% CI: 1.1-2.0), performance status (HR = 2.0, 95% CI: 1.5-2.5), stage at diagnosis (HR = 1.8, 95% CI: 1.3-2.3), and comorbidities (HR = 1.4, 95% CI: 1.0-1.8) were found to be independent predictors of survival, with hazard ratios greater than 1 indicating an increased risk of death. The univariate and multivariate analyses collectively identified smoking history, performance status, stage at diagnosis, and comorbidities as significant factors affecting prognosis in patients treated with bevacizumab biosimilar or reference bevacizumab (Shown as **Tables 3**, **4**).

**Table 3:** Univariate analysis of factors affecting prognosis.

Variable	p-value
Age	> 0.05
Sex	> 0.05
Smoking History	< 0.05
Performance Status (PS)	< 0.05
Stage at Diagnosis	< 0.05
Treatment Regimen (Biosimilar *vs*. Reference)	> 0.05
Comorbidities	< 0.05

**Table 4:** Multivariate analysis of factors affecting prognosis.

Variable	Hazard Ratio (HR)	95% Confidence Interval (CI)
Smoking History	1.5	1.1-2.0
Performance Status (PS)	2.0	1.5-2.5
Stage at Diagnosis	1.8	1.3-2.3
Comorbidities	1.4	1.0-1.8

These tables summarize the univariate and multivariate analyses conducted to identify factors that affect prognosis in patients treated with bevacizumab biosimilar or reference bevacizumab, specifically focusing on survival status.

### Safety

The occurrence of hypertension was not found to differ significantly between the two groups overall. Nevertheless, a more detailed analysis employing multivariate logistic regression modelling (adjusted for age, dose, etc.) revealed that biosimilars significantly increased the risk of hypertension in patients older than 70 years of age who were treated with a high dose of the drug, in comparison with the reference drug (P < 0.05). Nevertheless, there was no significant difference in the incidence of hypertension between the two groups following the exclusion of patients with a history of hypertension and those who had been treated with a very high dose. This indicates that the association between biosimilars and an increased risk of hypertension should be interpreted with caution, particularly in specific patient subgroups.

As indicated in **Table 5**, 76.42% of patients experienced treatment-related grade adverse events, with 808 out of 1058 patients experiencing such events. A total of 2,306 instances (23.06%) were observed in which three or more adverse events of grade 3 occurred. There were no fatalities. The frequency of transient adverse events (TEAEs) of any grade was comparable between the biosimilar and original drug groups, with the majority of adverse events falling into the grade 1 or 2 category. No statistically significant differences were observed in the occurrence of grade 3 and 4 TEAEs between the two groups. In the biosimilar group, the most common treatment-related adverse events (AEs) were fever (8.71%) and anaemia (9.15%). **Table 5** illustrates that the initial drug group exhibited the highest prevalence of anaemia, at 15.33%.

**Table 5:** Treatment-related adverse events (TEAEs) experienced by patients in the biosimilar (BG) and original drug (ODG) groups.

Adverse Event	BG (n=606)	ODG (n=452)
	Any Grade	Grade 3-4
	Total Patients	%
Fatigue	57	9.26%
Fever	47	7.91%
Pneumonia	8	2.27%
Anemia	70	9.15%
Leukopenia	17	2.93%
Neutropenia	58	8.87%
Thrombocytopenia	37	5.16%
Hypertension	38	3.34%
Bleeding	20	2.07%
Thromboembolism	4	0.43%

Adverse events are graded according to the Common Terminology Criteria for Adverse Events (CTCAE) v5.0.

Among the treatment-emergent adverse events (TEAEs), fever and anemia were the most commonly reported in the biosimilar group, affecting 8.71% and 9.15% of patients, respectively. In contrast, the original drug group showed a higher prevalence of anemia at 15.33%, while fever was less common. Other notable TEAEs included fatigue, pneumonia, leukopenia, neutropenia, thrombocytopenia, hypertension, bleeding, and thromboembolism, with varying frequencies across both groups. For instance, fatigue was seen in 9.26% of the biosimilar group and 10.90% of the original drug group, while hypertension was recorded in 3.34% of the biosimilar group and 5.12% of the original drug group.

## Discussion

### Key findings

The study confirmed the equivalence of the bevacizumab biosimilar and the original drug in terms of objective response rate (ORR) when used in combination with chemotherapy for NSCLC patients.

The biosimilar showed a slightly higher incidence of treatment-emergent adverse events (TEAEs), particularly in older patients with pre-existing hypertension receiving high doses, but overall, the adverse events were manageable.

Patient-specific factors such as age, comorbidities, and genetic profiles were identified as critical determinants of biosimilar safety and efficacy.

### Implications

The results suggest that bevacizumab biosimilars could be an effective alternative to the original drug in NSCLC therapy, potentially offering cost savings without compromising treatment outcomes.

The increased incidence of TEAEs in the biosimilar group warrants further investigation into patient subgroups to understand if there are specific populations at higher risk.

### Limitations and future directions

The retrospective analysis on bevacizumab and its biosimilar in NSCLC treatment reveals inherent constraints impacting interpretative accuracy, particularly concerning targeted therapies. The study’s retrospective nature introduces potential biases and confounding factors, complicating the balance of patient characteristics and treatment histories between the bevacizumab and biosimilar groups. Selection bias could arise from the non-representative sampling of the NSCLC population, notably affecting patients with specific genetic mutations. Varied treatment protocols and dosage intensities further cloud the isolation of individual drug effects. Absence of randomization risks compromising comparability between the original drug and biosimilar cohorts, potentially skewing outcomes. Short follow-up periods limit insights into long-term survival benefits, while exclusion criteria restrict applicability to broader patient subgroups, including those with prior treatments, concurrent therapies, brain metastases, and particular genetic mutations. The study highlighted improved outcomes with bevacizumab/biosimilar combinations and targeted therapies like EGFR-TKIs, yet mechanisms remain obscure, and other targeted therapies’ effects unexplored. Radiation status emerged as a significant factor affecting response rates, indicating complex interactions with bevacizumab and its biosimilar. Limited data on rare genetic mutation subgroups hinder robust conclusions on safety and efficacy. Adverse event reporting lacked depth on long-term impacts and quality of life considerations. These limitations necessitate cautious extrapolation of findings to clinical practice and call for future prospective, randomized trials to address gaps in understanding bevacizumab biosimilars’ role in NSCLC treatment, especially in conjunction with targeted therapies.

Retrospective study limitations, such as selection bias and incomplete data, necessitate the conduct of large-scale, prospective trials with rigorous monitoring to confirm safety and efficacy.

Future studies should include diverse patient populations to ensure findings are widely applicable and reveal treatment response variations.

Adaptive study designs and enhanced collaboration with healthcare communities are needed to improve inclusivity and precision in biosimilar therapy.

The study provides valuable insights into the safety and efficacy of bevacizumab biosimilars in NSCLC treatment, highlighting the importance of patient-specific factors in therapeutic decision-making.

Further research is essential to refine safety assessments, optimize patient benefit, minimize risks, and support evidence-based adoption of biosimilars in oncology.

## Conclusions

In summary, the retrospective analysis of 1058 patients with metastatic or locally advanced non-small cell lung cancer demonstrates that the bevacizumab biosimilar exhibits equivalent efficacy and safety profiles compared to the original bevacizumab. With comparable objective response rates and progression-free survival outcomes, the biosimilar offers a promising alternative treatment option. Although slight variations in the incidence of treatment-emergent adverse events were noted, these did not indicate a significant difference in overall safety. The study underscores the importance of monitoring hypertension risk, particularly in vulnerable subgroups. Given the limitations inherent to retrospective analyses, including potential selection bias, we advocate for larger, prospective trials to further validate these findings and explore the biosimilar’s effectiveness in patients with specific genetic variants. Such efforts will facilitate the advancement of precision medicine and promote equitable healthcare by ensuring the generalisability of treatment outcomes across diverse patient populations.

## Data Availability

The original contributions presented in the study are included in the article/supplementary material. Further inquiries can be directed to the corresponding author.

## Glossary

NSCLC: Non-Small Cell Lung Cancer – A type of lung cancer that includes several subtypes such as adenocarcinoma squamous cell carcinoma, and large cell carcinoma
ORR: Objective Response Rate – The percentage of patients who experience a partial or complete disappearance of the tumor in response to treatment
PFS: Progression-Free Survival – The length of time during and after the treatment of a disease, such as cancer, that a patient lives without the disease getting worse
TEAEs: Treatment-Emergent Adverse Events – Any new adverse event or worsening of a pre-existing condition that occurs after the start of a treatment
RECIST v1.1: Response Evaluation Criteria in Solid Tumors Version 1.1 – A set of published rules that define when cancer improves ("responds"), stays the same ("stabilizes"), or worsens ("progresses") during treatments with anticancer drugs
CTCAE v5.0: Common Terminology Criteria for Adverse Events Version 5.0 – A criterion used to grade the severity of adverse events that may be related to cancer treatments
